# Viral infection to the raphidophycean alga *Heterosigma akashiwo* affects both intracellular organic matter composition and dynamics of a coastal prokaryotic community

**DOI:** 10.1128/msystems.00816-25

**Published:** 2025-09-22

**Authors:** Hiroaki Takebe, Haruna Hiromoto, Tetsuhiro Watanabe, Keigo Yamamoto, Keizo Nagasaki, Ryoma Kamikawa, Takashi Yoshida

**Affiliations:** 1 Graduate School of Agriculture, Kyoto University 12918, Kyoto, Japan; 2 Faculty of Agriculture and Marine Science, Kochi University 12888, Nankoku, Kochi, Japan; 3 Research Institute of Environment, Agriculture and Fisheries, Osaka Prefecture 52741, Osaka, Japan; Ocean University of China, Qingdao, Shandong Province, China

**Keywords:** marine microalgae, prokaryotes, Algal virus, virocell, microcosm

## Abstract

Marine microalgae play a crucial role in the marine ecosystem by supplying dissolved organic matter to heterotrophic prokaryotes, which mediate the microbial loop. Microalgae are often infected by viruses, and in infected cells (virocells), the viruses modulate and often change the host metabolism for propagation and endo-metabolites. However, the impact of algal virocells on prokaryotic communities is not fully understood. In this study, we investigated whether lysates from virocells of *Heterosigma akashiwo,* a globally prevalent bloom-forming raphidophycean alga, cause shifts in prokaryotic community structure, and which metabolic compounds in the viral lysate might affect the surrounding prokaryotic populations. Using microcosm experiments, we cultured prokaryotic communities with a viral dissolved fraction (VDF) of *H. akashiwo*. Results revealed that certain prokaryotic populations assigned as *Vibrio* spp. pathogenic to fish and crustaceans grew specifically in response to the VDF. These *Vibrio* species possessed a gene module for branched-chain amino acids transporters, which were revealed to be enriched in VDF by gas chromatography-mass spectrometry analysis. Altogether, our findings suggest that viral infection-induced changes in biochemical properties of *H. akashiwo* cells can promote the growth of taxonomically and metabolically different prokaryotic populations, potentially impacting higher trophic-level consumers in marine ecosystems.

**IMPORTANCE:**

The primary production by marine microalgae and the consumption of the produced organic matter by prokaryotes significantly contribute to biogeochemical cycling. Microalgae are often infected by viruses, and in infected cells (virocells), the viruses modulate host metabolism for propagation and endo-metabolites. However, the impact of microalgal virocells on prokaryotic communities is not fully understood. This study investigates effects of lysates from virocells of *Heterosigma akashiwo*, a globally distributed harmful bloom-forming raphidophycean alga, on the prokaryotic community. Our data suggest that changes in biochemical properties in *H. akashiwo* virocells promoted the growth of specific bacterial populations that appeared to have metabolic capacity to utilize certain organic compounds enriched in the lysate. Additionally, as those populations included fish-pathogenic bacteria, we propose that viral infection to *H. akashiwo* can indirectly affect higher trophic-level consumers in marine ecosystems.

## INTRODUCTION

Marine microalgae significantly contribute to the primary production. Of the produced organic matters, the dissolved fractions (dissolved organic matter: DOM) are mainly consumed by planktonic heterotrophic prokaryotes (1–3). Viruses that infect these algae are also ecologically important as they contribute to the mortality of host cells and the release of organic matter from the cells, thereby affecting biogeochemical cycles (4, 5). Additionally, while particles of viruses floating in the seawater are regarded as those under a metabolically passive state, virus-infected host cells have been considered a “living form” of the viruses and thus referred to as virocells (6). Viruses infecting host cells actively modulate host cell metabolism through production of their daughter virions (6, 7).

The nucleo-cytoplasmic large DNA virus (NCLDV), a group of eukaryotic viruses, builds unique cytoplasmic structures, such as the viral factory and autophagosome, in their virocells. These structures lead to production of unique endo-metabolites that differ from those in uninfected cells (6, 8). For example, in virocells of the marine, bloom-forming haptophyte *Emiliania huxleyi,* the production of specific sphingolipids that are integral to viral particles is highly upregulated (9). Similar virocell-specific metabolic changes have been reported in the harmful bloom-forming pelagophyte *Aurerococcus anophagefferens* and the globally distributed pico-sized chlorophyte *Ostreococcus lucimarinus* (10, 11). Given that the preferred organic matter of heterotrophic prokaryotes varies among species (12, 13), virocells are likely to have different effects on prokaryotic community dynamics compared to the uninfected cells. Indeed, viral infection of *E. huxleyi* has been shown to cause significant shifts in natural prokaryotic communities in the mesocosm bags in coastal areas, though the specific virocell-derived organic matters were not analyzed in these studies (14).

 The raphidophycean alga *Heterosigma akashiwo* represents a crucial model for investigation of ecological relationships among microalgae, their viruses, and the surrounding prokaryotes. *H. akashiwo* is known for causing harmful blooms that result in mass mortality of fish and shellfish globally (15–18). Our previous study demonstrated that particular prokaryotic populations, such as those from *Alteromonadales* and *Vibrionales,* respond to DOM extracted from uninfected *H. akashiwo* cells in microcosm experiments (19). These responses differed from those observed with DOM from viral-free algal cells of a diatom, suggesting that blooms of different primary producers result in distinct prokaryotic community dynamics (20). Importantly, *H. akashiwo*-infecting viruses (HaVs) have been isolated during natural *H. akashiwo* blooms (21–23). Notably, up to 10^5^ cells of *H. akashiwo* per mL of surface seawater can disappear within 3 days, accompanied by an increase in extracellular HaV particles (23). If HaVs are the primary drivers behind the decline of *H. akashiwo* blooms, substantial amounts of organic compounds are expected to be released from *H. akashiwo* virocells during the final stages of the bloom. However, the impact of *H. akashiwo* virocells on surrounding prokaryotic community structures remains to be investigated.

In this study, we investigated how organic compounds derived from *H. akashiwo* virocells affect prokaryotic community dynamics. Organic compounds released from microalgal cells can be categorized into three different modes: exudates from healthy living cells, intracellular components released due to grazing, and viral-mediated cell lysates (24). To investigate these effects, we conducted microcosm experiments using a prokaryotic community collected from surface water in Osaka Bay. The experiments involved adding viral lysates of *H. akashiwo*, exudates, or intracellular components from uninfected *H. akashiwo*. We employed 16S rRNA gene amplicon analysis to assess prokaryotic community shifts and gas chromatography-mass spectrometry (GC-MS) to analyze DOM derived from both virocell and uninfected cells. Our findings revealed that virocell lysates, which had a unique organic matter composition, led to an increase in specific bacterial populations that preferentially appear to utilize these organic matters as substrates. Our data suggest that viral infection-induced changes in organic compounds may promote growth of prokaryotes with specialized metabolic capacities.

## RESULTS

### Abundance shifts of ***H. akashiwo*** and HaV during infection experiments

Since microalgal cell death occurs upon virus infection without the concomitant release of daughter virions (25), it is important to confirm that cell lysis is due to viral infection to obtain genuine viral lysates. In our study, cultures of *H. akashiwo* NIES-293 infected with HaV103, including those at a 10-fold dilution, exhibited growth arrest by day 3 (1.63 ± 0.18 × 10^5^ and 7.63 ± 1.16 × 10^4^ cells/mL, respectively) and subsequently lysed by day 6. In contrast, uninfected cultures continued to grow until day 14 (Fig. S1A). Extracellular viral DNA accumulation began as early as day 3 in the 10-fold diluted infected culture and peaked on day 5 (1.54 ± 1.22 × 10^7^ mcp copies/mL) (Fig. S1B), indicating the release of HaV particles. Therefore, the observed lysis of *H. akashiwo* NIES-293 was attributable to viral infection. These observations indicated that the resultant lysate was derived from genuine HaV-infected *H. akashiwo* virocells, which were used for subsequent experiments.

### Abundance shifts of prokaryotic cells in microcosms

**Fig 1:**
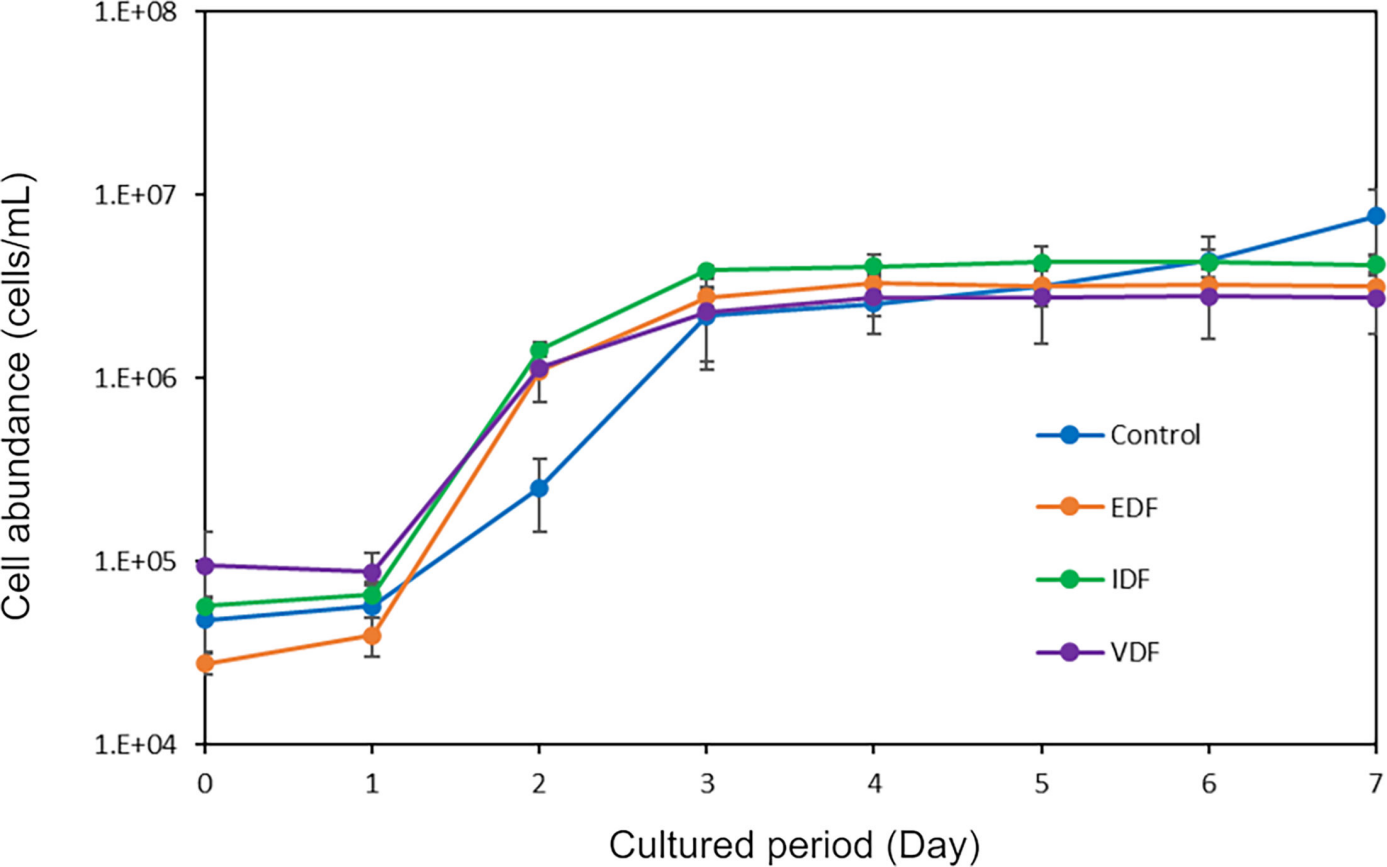
Shifts in abundance of prokaryotic cells during the microcosm experiment. Cell counts were obtained using flow cytometry. The average cell and viral numbers across triplicate flasks are shown. Error bars indicate standard deviation.

We compared the effects of viral lysate-, exudate-, and intracellular component-derived dissolved fraction of *H. akashiwo* NIES-293 (VDF, EDF, and IDF, respectively) on prokaryote cell numbers in the natural seawater from Osaka Bay. Cell densities across all treatments showed similar dynamics and abundances (Fig. 1). All treatments showed a sharp increase in cell densities from day 1 to day 3, reaching maximum densities on day 4 (2.56 ± 0.36 × 10^6^-4.08 ± 0.61 × 10^6^ cells/mL), followed by stagnation (Fig. 1). The cell densities in the VDF-treatment did not significantly differ from those in the EDF-treatment and IDF-treatment throughout the culture period, indicating that the effect of the *H. akashiwo* NIES-293 virocell lysate on prokaryotic abundance was comparable to that of intracellular components or exudates from uninfected *H. akashiwo* NIES-293 cells. These results indicate that certain prokaryotes grew in these conditions.

### hifts in prokaryotic community structure in response to VDF

Next, we investigated the effect of VDF on the marine prokaryotic community structure using 16S rRNA gene amplicon sequencing. From the original seawater sample, we identified 606 amplicon sequence variants (ASVs). During the microcosm experiments, the average number of ASVs obtained per flask was 1,080 for the control, 893 for the VDF treatment, 385 for the IDF treatment, and 976 for the EDF treatment (Table S1).

**Fig 2:**
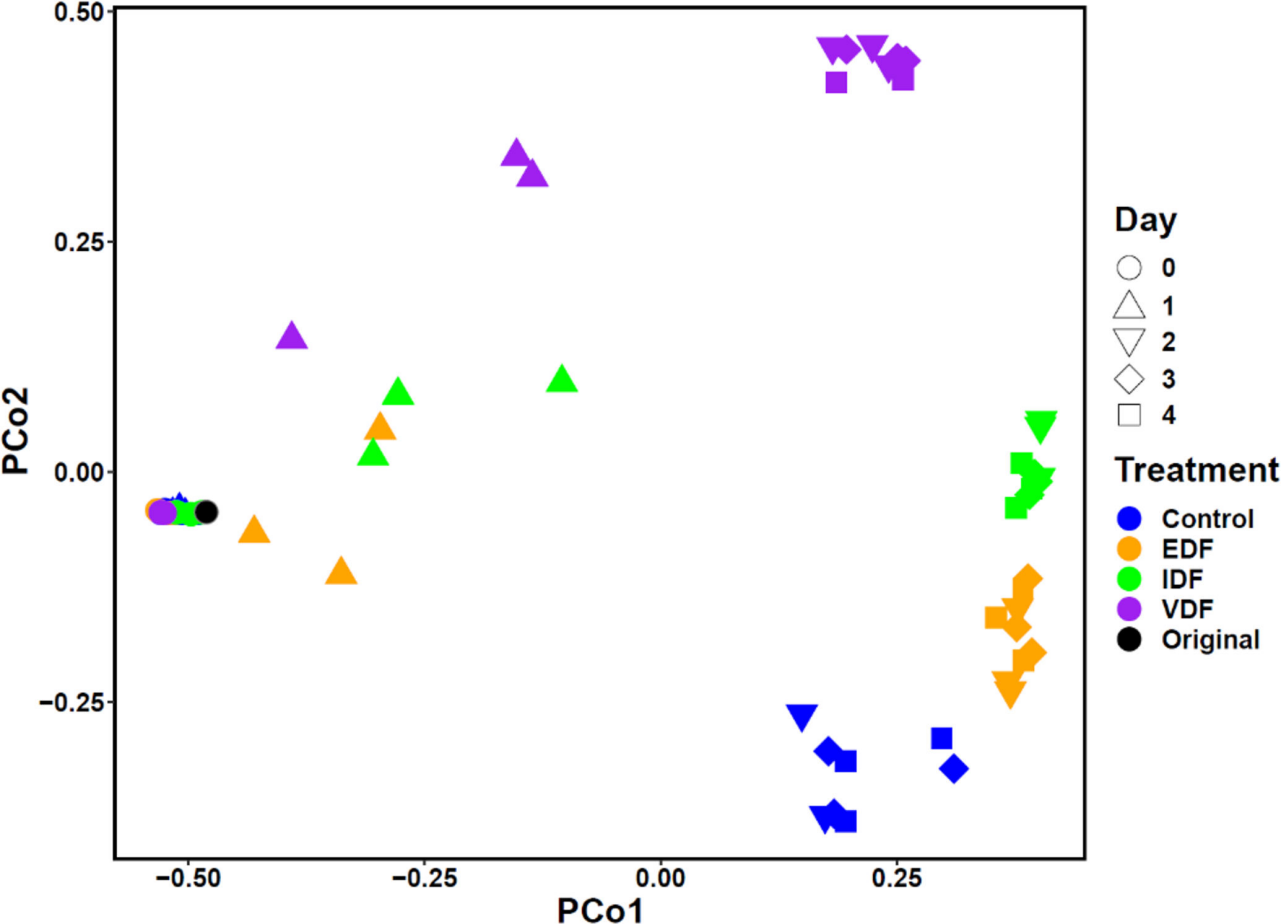
Comparison of ASV compositions across control, VDF-, IDF-, and EDF-treatments. The number of sequences of each sample was rarified into 3,453 reads prior to the analyses. Bray–Curtis dissimilarity among all samples was visualized using principal coordinate analysis (PCoA). Samples are distinguished by colors and shape according to their treatments and culture periods, respectively.

We examined whether the effect of VDF on ASV compositions differed from that of IDF, EDF, and f/2 medium (control) using principal coordinate analysis (PCoA) based on Bray-Curtis dissimilarity (Fig. 2). While day 0 samples were closely clustered with the original seawater sample, samples from day 2 to day 4 in the same treatments were more similar to each other but distinct from those in other treatments (ANOSIM, *P* < 0.05) and from day 0 samples within the same treatments (ANOSIM, *P* < 0.05) (Fig. 2). This PCoA indicates that prokaryotic community structures shifted differentially depending on the treatment and cultivation time. Those changes in the prokaryotic community structure might be influenced by the so-called "bottle effect," a well-documented phenomenon where confining seawater promotes the growth of opportunistic prokaryotes (26–29). However, even considering this effect, our results show that VDF from *H. akashiwo* differentially altered the prokaryotic community structure, leading to a distinct profile compared to that of IDF and EDF. This suggests that the quality of organic matter is a key factor in shaping the microbial community. The difference in the ASV composition of the control compared to the original seawater sample is considered to be due to the presence of organic matter and nutrients in the f/2 medium we used, which likely had a different impact on the dynamics of prokaryotes.

We further analyzed which phyla (class level for Proteobacteria) became dominant in the VDF treatment (Fig. S2). On day 0, the dominant heterotrophic prokaryotes in the VDF samples were Alphaproteobacteria and Gammaproteobacteria (24.5% and 24.7%, respectively), followed by Bacteroidetes (15.2%), whereas smaller proportions were of Actinobacteria, Verrucomicrobia, and Thermoplasmatota. Alphaproteobacteria, Gammaproteobacteria, and Bacteroidetes remained abundant throughout the microcosm experiment, while their relative abundances shifted during the experiment. By day 2, Gammaproteobacteria dominated almost entirely, while the proportions of Alphaproteobacteria and Bacteroidetes decreased. The increase in the total prokaryotic cell count in the VDF-treatment (Fig. 1) suggests that lysates from *H. akashiwo* NIES-293 virocells specifically promoted the growth of certain populations in Gammaproteobacteria populations (Fig. S2). Similar shifts in community structure were also observed in EDF and IDF treatments, although the proportions of dominant phyla or classes varied (Fig. S2). In contrast, Campylobacterota became more dominant in the control treatment supplemented with f/2 medium. These shifts at the phylum- and class-level were more explicit from day 2 and consistent with the PCoA patterns (Fig. 2). Additionally, although Cyanobacteria accounted for 22.2% (VDF treatment)–36.2% (IDF treatment) on day 0, their abundance declined by day 1 and was barely detectable by day 2. This suggests that the Cyanobacteria present in the original seawater sample perished shortly after the start of the incubation. Consequently, new organic matter production by autotrophic bacteria likely had a minimal influence on the dynamics of heterotrophic prokaryotes.

### Abundant ASVs in VDF and other treatments

Although the PCoAs indicate distinct community structure shifts among VDF, EDF, and IDF treatments, the phylum- or class-level proportions were not largely distinct across the treatments (Fig. S2). This suggests that the differences in community structure would be observed at finer taxonomic levels. Therefore, we focused on prokaryotes that specifically responded to and grew on VDF at the ASV level. Since changes in relative abundance during cultivation do not necessarily reflect cell growth and death, we analyzed the dynamics of abundant ASVs by calculating their approximate cell density, which combines the total cell number and relative abundance.

**Fig 3:**
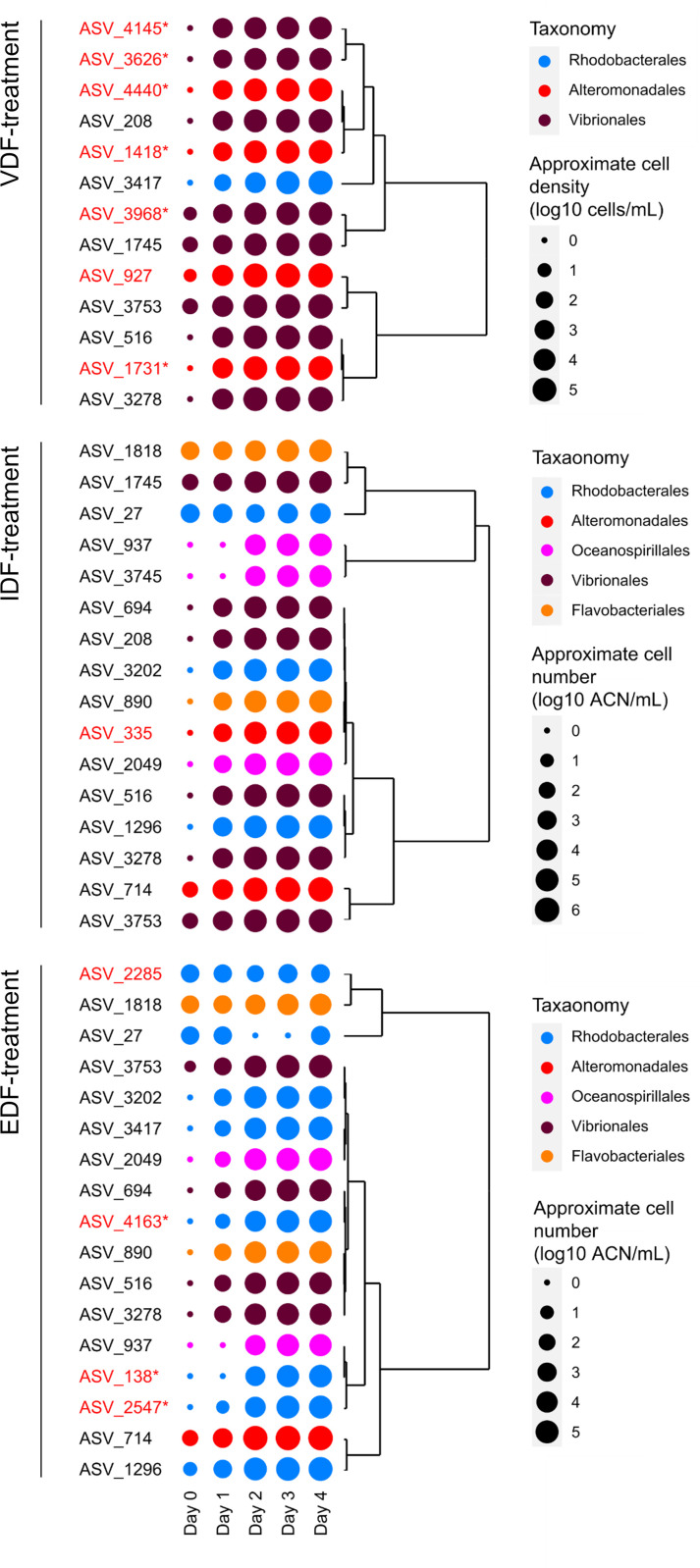
Dynamics of ASVs abundant in VDF-, IDF-, and EDF treatment. The plots show average approximate cell density in the triplicate flasks on a log scale. Colors show order-level taxonomy of each ASV. Dendrograms represent similarity in approximate cell density dynamics among ASVs. ASVs abundant exclusively in VDF-, IDF-, or EDF-treatments are highlighted in red, with treatment-specific ASVs indicated by asterisks.

We identified 13, 16, 17, and 11 abundant ASVs in VDF-, IDF-, and EDF-treatments, and the control, respectively (Fig. S3). Of the 13 ASVs abundant in the VDF treatment (Fig. 3), 7 were only abundant in the VDF treatment. These included three ASVs from the Vibrionaceae family (ASV_3626; genus not assigned, ASV_3968; *Vibrio*, and ASV_4145; *Vibrio*), three ASVs from the genus *Pseudoalteromonas* (ASV_1731, ASV_4440, and ASV_927), and one ASV from *Psychrobirium* (ASV_1418) (Fig. 3; Table S2). All these ASVs belong to Gammaproteobacteria. Differential abundance testing using Lefse indicated that all these ASVs, except for *Pseudoalteromonas* ASV_927, were significantly more abundant in the VDF treatment compared to the other treatments (Table S2). Hereafter, the six ASVs significantly more abundant in the VDF treatment were referred to as VDF-specific ASVs.

The remaining six abundant ASVs in the VDF treatment were also found to be abundant in at least one of the other treatments (Fig. S3; Table S2). These shared ASVs were assigned to the genera *Thalassibius* (ASV_3417) and *Vibrio* (the other ASVs) based on the Silva database (Table S1). Additionally, nine ASVs abundant in either or both of IDF and EDF were considered capable of utilizing dissolved fractions from uninfected *H. akashiwo* cells. Among these, *Pseudoalteomonas* ASV (ASV_335) and four of Rhodobacteraceae ASVs (ASV_138; genus not assigned, ASV_2285; *Planktomarina*, ASV_2547; genus not assigned, and ASV_4163; Octadecabacter) were abundant exclusively in the IDF- and EDF-treatment, respectively (Table S2). The comparison of abundant ASVs highlighted the distinct effects of dissolved fractions from virocells and uninfected *H. akashiwo* cells on prokaryotic community structures at the ASV level, even within the same genera. Hereafter, ASV_927, shared ASVs, and ASVs abundant in the treatments other than VDF will be referred to as VDF-nonspecific ASVs.

### Taxonomic and phylogenetic relationships between VDF-specific and -nonspecific ASVs

As most of VDF-specific and -nonspecific abundant Vibrionaceae ASVs (3 and 6 ASVs, respectively) were identified as belonging to the genus *Vibrio*, we mapped these ASVs onto a reference phylogenetic tree of *Vibrio* spp. to reveal phylogenetic relationships. The three VDF-specific ASVs were not closely related to each other and were assigned to distinct clades, termed Vv1–Vv3 (Fig. S4). However, since these phylogenetic positions are based on mapping partial sequences to a full-length reference phylogenetic tree, the correspondence between these ASVs and the phylogenetically related species is still unclear. Therefore, we further surveyed for their closely related species based on the nucleotide sequence with 100% identity (30). ASV_3968 of Vv2 and ASV_4145 of Vv3 had identical nucleotide sequences to 16S rRNA genes from *Vibrio penaecida* and of *V. galatheae, V. hyugaensis, V. alginolyticus, V. harveyi,* and *V. nartriegens* (Table 1). Specifically, the Vv2 clade contained *V. penaecida*, while the Vv3 clade included *V. alginolyticus*, *V. harveyi*, and *V. natriegens* (Fig. S4).

**TABLE 1:** Phylogenetic and taxonomic assignment of VDF-specific and -nonspecific ASVs^*a*^

ASV	Clade	ASV_ID	Closest isolate	Accession ID(s) of the closest isolate
*Vibrio*	Nv1	ASV_516	*Vibrio gigantis*	NR_114910
		ASV_694		
		ASV_1745		
	Nv2	ASV_208		
		ASV_3278		
		ASV_3753	*Vibrio chagasii*	NR_117891
	Vv1	ASV_3626		
	Vv2	ASV_3968	*Vibrio penaeicida*	NR_042121, NR_113790
	Vv3	ASV_4145	*Vibrio galatheae*	NR_147758
		ASV_4145	*Vibrio hyugaensis*	NR_145569
		ASV_4145	*Vibrio alginolyticus*	NR_044825, NR_117895, NR_122060
		ASV_4145	*Vibrio harveyi*	NR_043165, NR_113784
		ASV_4145	*Vibrio natriegens*	NR_026124, NR_113786, NR_117890
*Pseudoalteromonas*	Na1	ASV_927	*Pseudoalteromonas phenolica*	NR_113299
	Na2	ASV_335	*Pseudoalteromonas marina*	NR_042981
	Va	ASV_1731		
		ASV_4400		

^*a*^ Regarding ASVs whose sequence showed 100% identity with cultured bacteria deposited in the RefSeq NR database, the hit bacterium is indicated with the corresponding accession number.

The five VDF-nonspecific abundant *Vibrio* ASVs were grouped into two clades, termed Nv1 and Nv2 (Fig. S4). Nv1 clade included ASV_516, ASV_694, and ASV_1745, while Nv2 clade included ASV_208, ASV_3278, and ASV_3753 (Table 1). ASV_516 (Nv1) and ASV_3753 (Nv2) show 100% nucleotide sequence identity with *V. gigantis* and *V. chagasii*, respectively (Table 1), suggesting that multiple ASVs within Nv1 and Nv2 are intraspecies populations of *V. gigantis* and *V. chagasii*, respectively. Due to the absence of 16S rRNA gene sequences longer than 1,500 bp for *V. gigantis* and *V. chagasii* in public databases, these species were not included in the reference phylogenetic tree (Fig. S4).

The VDF-specific and VDF-nonspecific *Vibrio* spp. possess distinct ecological characteristics. *V. penaecida* (Vv2) and *V. harveyi* (Vv3) are known pathogens of tiger prawn (31) and bony fish such as sea bream and amberjack (32, 33), respectively. *V. gigantis* (Nv1) is an opportunistic pathogen of European seabass (34) and has been shown to respond to intracellular components of uninfected *H. akashiwo* cells in our previous study (20). *V. chagasii* (Nv2) is a known pathogen of oysters and mussels (35, 36). Although no identical nucleotide sequences to ASV_3626 (Vv1) are available in the database, its mapped position suggests a closer relation to *Vibrio profundi* TP187, originally isolated from a seamount in the tropical western Pacific (37) (Fig. S4).

Similarly, the VDF-specific abundant Pseudoalteromonadaceae ASVs (ASV_1731 and ASV_4400; *Pseudoalteromonas*) clustered together in a single clade (Va), distinct from the positions of the VDF-nonspecific Pseudoalteromonadaceae ASVs in the reference phylogenetic tree (Fig. S5). The VDF-specific ASVs are closely related to *Pseudoalteromonas aliena* or *Pseudoalteromonas arctica* (Fig. S5), although no identical 16S rRNA gene sequences to these species are available. In contrast, two VDF-nonspecific ASVs, ASV_927 (Na1) and ASV_335 (Na2), were placed in separate clades. The Na1 clade was mapped to two distinct branches (Fig. S5), likely because the ASV sequences were shorter than the near full-length 16S rRNA gene sequences used to build the reference phylogenetic tree, which hindered accurate placement. Therefore, as with the Vibrionaceae ASVs, we did not perform phylogenetic comparisons with isolates based on tree placement. Instead, we identified closely related strains based on 100% nucleotide identity. ASV_335 and ASV_927 showed 100% nucleotide sequence identity with 16S rRNA genes of *Pseudoalteromonas marina* and *P. phenolica*, respectively (Table 1). The Na1 clade included *P. phenolica*. Additionally, the sequence of *P. marina* (CP023558) clustered with CP019162 *Pseudoalteromonas* sp. with >99.7% sequence identity, and CP019162 was included in the Na2 cluster, indicating *P. marina* also belonged to Na2 (Fig. S5). *P. marina* is a marine species known for forming biofilms that facilitate the settlement of *Mytilus coruscus* larvae (38) and responding to intracellular components of uninfected *H. akashiwo* cells (20). *P. phenolica* is noted for producing an antibiotic against certain bacteria (39).

For the *Psychrobirium* ASVs, only the VDF-specific lineage (ASV_1418) was identified in our experiment, so mapping to a reference phylogenetic tree of closely related species was not performed. However, ASV_1418 was found to be identical to 16S rRNA gene sequences of *Psychrobium conchae*. Phylogenetically close relatives of this species were found to be associated with sea bream eggs, although their pathogenicity has not been reported (40).

**Fig 4:**
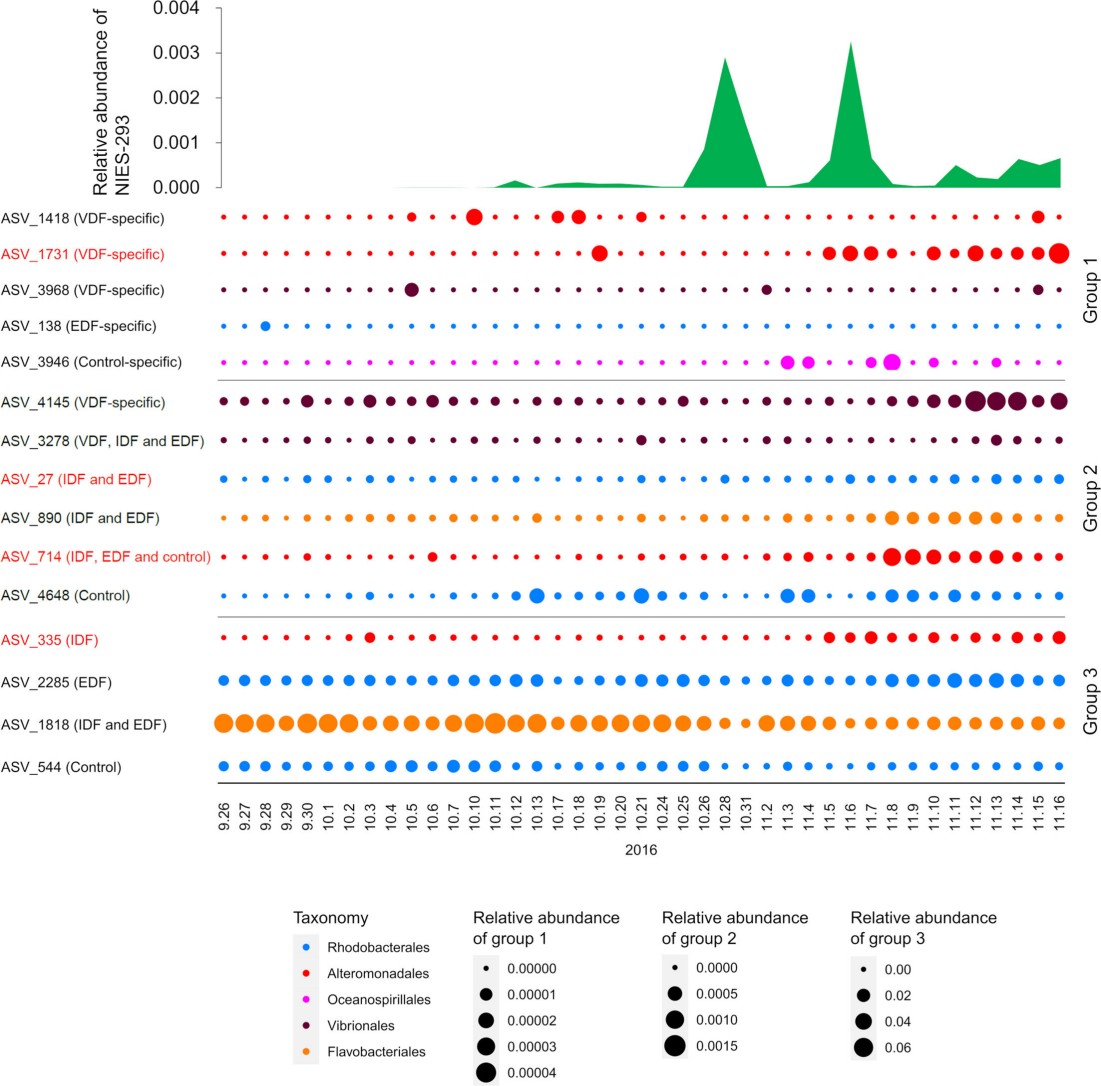
Co-occurrence dynamics of the *H. akashiwo* and the abundant ASVs during the microcosm experiments in the natural bloom samples. The microbiome sequence data sets almost daily collected between September 26 and November 16, 2016 (41) were used. Relative abundance of *H. akashiwo* NIES-293 close relatives was calculated by mapping quality-controlled reads of 18S rRNA genes to the NIES-293 sequence with 97% identity using VSEARCH. Relative abundance of each ASV was calculated by mapping quality-controlled reads of 16S rRNA genes to the ASV sequence with 100% identity using VSEARCH. These ASVs are divided into three groups according to the range of relative abundance and displayed with different legends. The ASVs that showed a significant positive correlation with the dynamics of *H. akashiwo* NIES-293 close relatives are highlighted in red (Spearman correlation; *r* > 0.6, *P* < 0.01 and *Q* < 0.05).

It is important to observe whether the association of these abundant ASVs with *H. akashiwo*-derived compounds observed in microcosm experiments also occurs in natural environments. We investigated the dynamics of VDF-specific and VDF-nonspecific abundant ASVs in natural bloom samples containing *H. akashiwo* collected in Monterey Bay, USA (41). In the microcosm experiment, VDF-specific ASVs became dominant approximately 2 days after the addition of organic matter. Therefore, a time delay of 2 sampling days was allowed in the analysis. We found a significant positive correlation between the relative abundance of VDF-specific *Pseudoalteromonas* ASV_1731 (Va) and VDF-nonspecific *Pseudoalteromonas* ASV_335 (Na2) with that of *H. akashiwo* (Table S3). *H. akashiwo* increased during October 26–31 and November 5–7, 2016, and both ASV_335 and ASV_1731 also increased on 5 November 2016 (Fig. 4). Importantly, the dynamics of ASV_1731 did not exhibit temporal autocorrelation during this period (*P* < 0.05) (Fig. S6), suggesting that it is highly likely correlated with the bloom of *H. akashiwo*. ASV_335 showed partial autocorrelation at a maximum of two sampling intervals (Fig. S6). Although VDF-specific *Vibrio* ASVs, ASV_3968 and ASV_4145, as well as VDF-specific *Psychrobium* ASV_1418, were detected during the bloom, the correlation between their dynamics and *H. akashiwo* was not statistically significant (Table S3). Among lineages showing no correlation with the VDF in the microcosm experiments, Rhodobacteraceae ASV_27 (abundant in IDF and EDF) and Alteromonadaceae ASV_714 (abundant in control, IDF, and EDF) showed positive correlations with the relative abundance of *H. akashiwo* (Fig. 4). However, both ASVs exhibited partial autocorrelation during the sampling period (Fig. S6), suggesting that their association with *H. akashiwo* blooms is not definitive.

### Difference in metabolic capacity between Vv clade and Nv clade

To gain insights into the metabolic capacities underpinning the increase of VDF-specific populations, we performed a comparative genomics analysis of the close relatives (100% nucleotide identity of 16S rRNA sequences) of VDF-specific ASVs based on the composition of KEGG modules (42). We excluded *Psychrobirium* and *Pseudoalteromonas* from further analyses as no VDF-nonspecific ASVs were found for *Psychrobirium*, and only one genome was available for close relatives of VDF-specific and VDF-nonspecific ASVs within *Pseudoalteromonas*. Consequently, we focused on *Vibrio* spp., which included close relatives of both VDF-specific (Vv2 and Vv3 clades) and VDF-nonspecific (Nv1 and Nv2 clades) ASVs, with available genomes (5, 44, 2, and 2 genomes, respectively) (Table S4). Note that no genome was available for the close relative of the Vv1 ASVs. A total of 248 KEGG modules were identified in at least one genome, with varying patterns of module presence among the four clades (Table S5). In particular, five modules were frequently present (> 70%) in the close relatives of Vv2 and Vv3 ASVs (46 genomes), but not detected in those of the Nv clades ASVs (Table S5), suggesting potential different metabolic capability between Vv clades and Nv clades. The specific presence of these functional modules in genomes of close relatives of the Vv clades was also supported by statistical analysis (Mann–Whitney *U* test: *P* < 0.005). These modules included (i) biosynthesis of ectoine (M00033), an osmolyte; (ii) branched−chain amino acid transport system (M00237) for branched-chain amino acid acquisition; (iii) phosphotransferase (PTS) system cellobiose−specific II component (M00275) for importing and phosphorylating cellobiose; (iv) nitrate/nitrite transport system (M00438) for nitrogen source acquisition; (v) CreC−CreB (phosphate regulation) two-component regulatory system (M00449) for environmental condition recognition, such as carbon supply and oxygen availability (43, 44), and specific cellular activities, such as biofilm formation (45) and motility (46) (Table S5). The branched-chain amino acid transport system was exclusive to the close relatives of Vv2 and Vv3 ASVs, while the valine/isoleucine biosynthesis module (M00019) was present in genomes across those of all the four clades ASVs, indicating that those branched-chain amino acids might be biosynthesized but not essential amino acids (Table S5). Furthermore, over 97% of the close relative genomes (*n* = 44) belong to Vv3 ASVs encoded the leucine degradation module (M00036), which converts leucine into acetyl-CoA, potentially linking it to the branched-chain amino acid transport system (Table S5). Some of those modules were also detected in genomes of other *Vibrio* populations corresponding to ASVs that were not abundant in any treatment (Table S6).

### Metabolites accumulated in VDF

**Fig 5:**
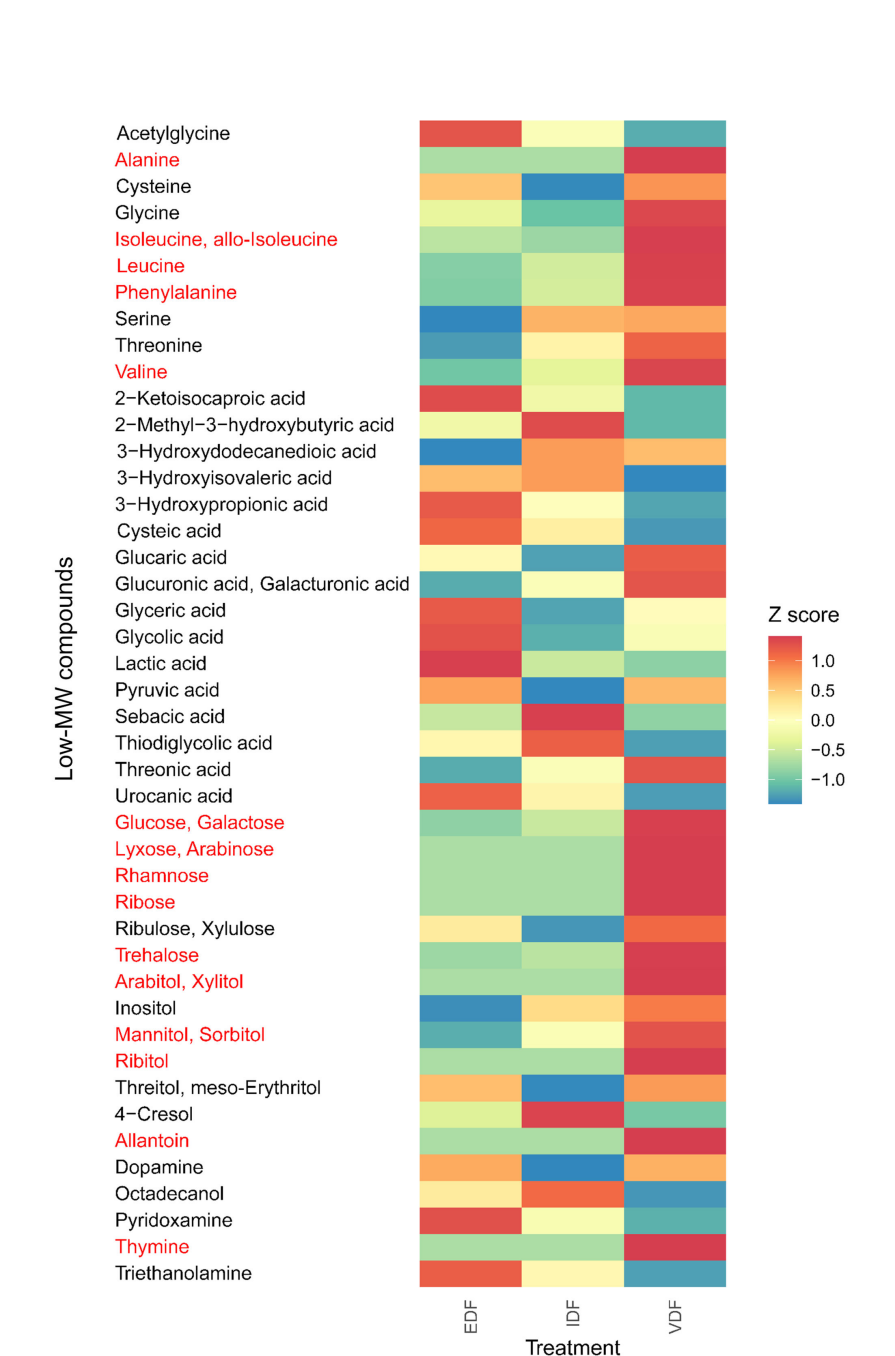
Comparison of relative abundance of low-molecular weight (MW) compounds enriched in VDF among VDF, IDF, and EDF. Only low-MW compounds that showed higher relative abundance in any of VDF, IDF, or EDF than in control are presented. The low-MW compounds that were at least twice as abundant in VDF than in IDF and EDF are shown in red. The relative abundance of each compound was calculated by dividing its peak area by that of internal control (2-isopropylmalate). The relative abundance was normalized with the carbon concentration of each sample, enabling comparison between samples of different carbon concentrations. The normalized relative abundance of each compound in each treatment was converted to z-score.

We investigate whether the organic matter composition in VDF includes substrates specific to the metabolic capacities of the Vv clades using GC-MS analysis. Compared to the composition of low molecular weight (MW) compounds in f/2 medium, we detected 44 low MW compounds that were significantly more dominant in either of the three treatments (Table S7). These compounds were likely derived from *H. akashiwo* cells rather than from the medium. The relative abundance of these 44 compounds varied among the different DFs (Fig. 5). Especially, 15 of these compounds were at least twice as abundant in VDF as in the other DFs, suggesting a specific enrichment of these 15 compounds in the VDF (Fig. 5).

The 15 low-MW compounds enriched in the VDF can be divided into four types: amino acids (alanine, isoleucine/allo-isoleucine, leucine, valine, and phenylalanine), sugars (glucose/galactose, ribose, lyxose/arabinose, rhamnose, and trehalose), sugar alcohols (mannitol/sorbitol, arabitol/xylitol, and ribitol), and nucleotide derivatives (thymine and allantoin) (Fig. 5). Compounds separated by a slash represent those that could not be distinguished by GC-MS. Notably, all the branched-chain amino acids were specifically enriched in the VDF.

## DISCUSSION

In this study, we demonstrated that lysates released from *H. akashiwo* virocells promoted the growth of different prokaryotic populations compared to intracellular components or exudates from uninfected *H. akashiwo* cells (Fig. 2 and 3). The virocell-specific prokaryotic populations included ASVs from the genera *Vibrio*, *Pseudoalteromonas*, and *Psychrobirium* (Table S2). While our previous microcosm experiments showed that certain populations in these genera responded to uninfected *H. akashiwo* cells (19, 20), this study revealed that the preference for *H. akashiwo* virocells or uninfected cells varied even among ASVs within the same genera (Table 1). Interactions between *H. akashiwo* virocells and certain prokaryotic populations, and those between uninfected *H. akashiwo* cells and other prokaryotic populations, likely occur not only under laboratory conditions but also in the natural environments. This is supported by the observed correlations between the dynamics of VDF-specific and VDF-nonspecific *Pseudoalteromonas* populations and *H. akashiwo* in Monterey Bay, US (Fig. 4). Although the correlation with *H. akashiwo* dynamics was not statistically significant for VDF-specific and VDF-nonspecific *Vibrio* populations, these groups were also present during the bloom. Given the detectable concentrations of HaV during *H. akashiwo* blooms in previous studies, it is likely that *H. akashiwo* cells in natural environments are exposed to HaV infection, leading to the lysis of at least some of the cells (23). Therefore, natural *H. akashiwo* blooms are likely a mixture of uninfected cells and virocells, and increases in both VDF-specific and VDF-nonspecific populations in natural blooms would be rational. However, the proportion of *H. akashiwo* virocells in natural blooms remains unknown as there is currently no established method to quantitatively distinguish between virus-infected and uninfected *H. akashiwo* cells. This remains a subject for future research.

Virocell-specific prokaryotic responses have been reported in several eukaryotic microalgae (47) and cyanobacteria (48). Experiments using ^13^C-labeled tracers or quantification of DOM in liquid media suggest that DOM from the virocells is utilized by specific, heterotrophic bacteria (47). However, the specific compounds from microalgal virocells that promote the growth of specific prokaryotic communities remained unclear in these studies. Genomic analyses of *Vibrio* spp., which are closely related or phylogenetically similar to the VDF-specific *Vibrio* ASVs (ASV_3968 and ASV_4145), reveal that these strains specifically encode a branched-chain amino acid transport system (M00237) not detected in the close relatives of VDF-nonspecific *Vibrio* populations (Table S5). Additionally, these strains also encode a valine/isoleucine biosynthesis module (M00019) (Table S5). The biosynthesis of valine and isoleucine begins with pyruvate, a key metabolite involved in the biosynthesis of fatty acids, lipids, and isoprenoids. Consequently, the biosynthesis of branched-chain amino acids might compete with the biosynthesis of other biologically essential compounds. Although acquisition of branched-chain amino acids from the environments consumes ATP since the transporter belongs to the ATP binding cassette family (49), this system might provide an additional source of amino acids, thereby conserving pyruvate in VDF-specific *Vibrio* populations. Furthermore, all genomes of Vv3 close relatives, except one, encode leucine degradation modules (M00036) (Table S5). This pathway degrades one of the branched-chain amino acids into acetyl-CoA, which is also a key substrate used for various metabolic pathways, including fatty acid biosynthesis and respiration. Therefore, acquiring branched-chain amino acids from VDF could support the synthesis of various compounds or energy conservation in the Vv3 clade, promoting its growth. Notably, the relative abundances of branched-chain amino acids, such as leucine, isoleucine, and valine, were higher in the VDF than in the IDF and EDF (Fig. 5). These VDF-specific compounds may serve as substrates for the branched-chain amino acid transport system in the *Vibrio* populations. This is the first report to identify candidate metabolic compounds from virocells that might affect the surrounding prokaryotic community structure.

 Other metabolic features in prokaryotic populations might also influence their dynamics in the presence of *H. akashiwo* virocell-derived compounds. In addition to the branched-chain amino acid transport system, four additional metabolic modules were prevalent in the close relatives of Vv populations: ectoine biosynthesis, phosphotransferase system, cellobiose-specific II component, nitrate/nitrite transport system, and creC−creB two-component regulatory system (Table S5). Especially, creC, a sensory kinase in the creC-creB regulatory system, is known to respond to changes in carbon supply (43, 44). Sugars such as glucose/galactose, ribose, lyxose/arabinose, rhamnose, and trehalose, analyzed by GC-MS, were relatively enriched in *H. akashiwo* VDF when compared to other DFs. Changes in the composition of carbon sources due to *H. akashiwo* virocell lysis might be sensed by creC kinase, potentially triggering a response from the creB regulatory factor, affecting activities of VDF-specific *Vibrio* populations, such as biofilm formation and motility (44, 46). Currently, we have not identified plausible links of virocell-derived compounds and the modules for ectoine biosynthesis, phosphotransferase system, cellobiose-specific II component, and nitrate/nitrite transport system. Further research is needed to elucidate these potential connections.

However, there are several limitations in linking the organic matter derived from *H. akashiwo* virocells to the metabolic functions of VDF-specific *Vibrio* populations as follows. i) It is important to recognize that the abundance of prokaryotic population is determined by complex interplay of factors, including both nutrients and environmental conditions. Thus, the increase in specific populations cannot be attributed to a single factor alone. While *H. akashiwo* virocell-derived VDF may regulate specific *Vibrio* populations, it does not necessarily promote the increase of all *Vibrio* populations corresponding to the strains with aforementioned metabolic modules including the branched-chain amino acid transport system in the present microcosm experiment (Table S6). It remains possible that the less abundant populations could become more prevalent and abundant under different conditions with VDF-derived compounds. ii) The genomic information compared in this study was not derived from the prokaryotes that appeared in the microcosm experiment, but was instead based on an extrapolative approach using publicly available data extracted based on 16S rRNA gene sequences. Therefore, if the modules enriched in the close relatives of the Vv2 and Vv3 populations were not vertically inherited but instead acquired through horizontal gene transfer in those phylogenetic groups, it is possible that those modules are absent in the genomes of the VDF-specific populations detected in this experiment. iii) Although the composition of the organic matter added at the start of the microcosm experiment was analyzed, it was not analyzed during or at the end of the incubation. Therefore, the compounds found to be abundant at the beginning may have been transformed through consumption by the prokaryotic community, and the influence of those compounds on the dynamics of the VDF-specific ASVs could involve both direct and indirect effects. iv) Metabolomic analysis could only be performed in singlicate. As a result, it remains unclear whether the enrichment of specific organic matters such as branched-chain amino acids occurs only under specific conditions or whether they are consistently enriched in the lysate regardless of the environmental condition during infection. All the possibilities described above should be addressed by combining whole-genome analysis and stable isotope-labeled metabolomic analysis with similar culture experiments conducted under diverse environmental conditions in the future.

The effects of some low-MW compounds enriched in the virocell lysates on viral particle production remain unclear. Viral particle production requires nucleotides and amino acids for DNA/RNA and protein synthesis, respectively. So far, it has been reported that the major capsid protein of *Chlorella* viruses, which infect certain strains of the green alga *Chlorella*, is modified by glycans (50). However, no studies have linked sugar alcohols to the production of microalgal viruses. Amino acids, sugars, sugar alcohols, and nucleotide derivatives are likely biosynthesized by some algal cells as they have been detected in cellular compounds, mucilage sheaths, and/or excreted compounds from various algal cells (9, 51–58). However, lyxose and allantoin are exceptions. Allantoin, an intermediate metabolite in purine catabolism and common in plants (59), has not been reported in algae. To confirm this, we searched for homologous proteins involved in *Arabidopsis* allantoin biosynthesis from xanthin (Accession nos. AT4G34890, AT4G34900, AT2G26230, and AT5G58220) in the transcriptome data of uninfected *H. akashiwo* CCMP452 (https://dx.doi.org/10.6084/m9.figshare.3840153.v3), but found no homologs. Similarly, homologous proteins were not found in the genome of HaV01 (https://www.ncbi.nlm.nih.gov/datasets/taxonomy/97195/). Whether the virocell-specific low-MW compound identified as allantoin is indeed genuine allantoin or an as-yet-unknown compound that is difficult to be distinguished from allantoin remains to be confirmed. Future studies should clarify whether it is true allantoin and elucidate its role, if any, in viral particle production within *H. akashiwo* virocells.

Finally, the virocell-specific *Vibrio* populations or their close relatives are pathogens of tiger prawns and bony fish including sea bream and amberjack, while virocell-nonspecific ones infect oyster and mussel (Table 1). Therefore, changes in prokaryotic dynamics caused by viral infections in *H. aksahiwo* might lead to negative impacts in marine environments. Actually, blooms of *H. akashiwo* are known to damage sea bream, amberjack, and oysters (60, 61). Proposed mechanisms for these impacts include excessive mucous secretion and production of reactive oxygen species, organic toxin, and hemolytic compounds from *H. akashiwo* cells during blooms; however, these mechanisms remain to be elucidated (61). Although not reported previously, these negative impacts might be indirectly mediated by *H. akashiwo*, possibly through bacteria that respond to the alga. The biological and biogeochemical links between viral infections in *H. akashiwo* and the marine material cycling and dynamics of higher trophic-level consumers, such as fish and crustaceans, could be significant.

In the present study, we investigated effects of lysates from *H. akashiwo* virocells on the dynamics of coastal prokaryotic populations. Our data suggested that the virocell-specific prokaryotes had certain bacterial populations that may have potential to utilize specific organic matters enriched only in virocells, indicating that changes in organic compounds induced by viral infection promote growth of prokaryotes with specific metabolic capacities. Additionally, as the population included pathogenic bacteria of fish or crustacean, viral infection to *H. akashiwo* can indirectly affect the dynamics of higher-trophic level consumers. Considering that *H. akashiwo* is a widely distributed bloom-forming species and viral infection is a crucial factor in terminating their blooms, understanding these dynamics is crucial for assessing the broader ecological impacts of *H. akashiwo* blooms and their associated viral infections.

## MATERIALS AND METHODS

### Culturing of* H. akashiwo*

*H. akashiwo* strain NIES-293 was purchased from the National Institute for Environmental Studies (NIES) and was maintained axenically in the artificial seawater Marine Art Hi (Tomita Pharmaceutical Co.) supplemented with the components of f/2 medium (62) at 20°C with a 12 hour light/12 hour dark photoperiod at an irradiance of 40 µmol photons m^−2^ s^−1^. The culture was grown in 1.5 L of the medium under these conditions. It was then divided into three portions (500 mL, 600 mL, and 400 mL) for the following purposes: extraction of exudates and intracellular components from uninfected cells, preparation of viral lysates, and monitoring of growth until the stationary phase, respectively.

### Collection of exudates and intracellular components

To prepare intracellular components and exudates from uninfected cells, 500 mL of the culture was centrifuged at 1,500 × *g* for 5 min using a High Capacity Bench-top Centrifuge LC-220 (TOMY SEIKO). The resulting supernatant was filtered through a 0.2 µm pore size PVDF filter (Millipore), and the filtrate was designated as the exudate-derived dissolved fraction (EDF). Intracellular components were extracted from the pellet using the method outlined in our previous study (20) yielding the intracellular component-derived dissolved fraction (IDF).

### Viral infection experiments of ***H. akashiwo***

The *H. akashiwo*-infectious virus strain HaV103, isolated from Uranouchi Bay, Kochi, Japan, in 2021, was inoculated in 600 mL of the NIES-293 culture with an initial multiplicity of infection (MOI) of approximately 0.008, to prepare viral lysates. Sixty mL of the infected culture was reserved for monitoring the dynamics of NIES-293 and HaV103. After the culture was decolorized, viral lysates were harvested from the remaining 540 mL. The supernatant was filtered through a 0.2 µm PVDF filter (Millipore). The resulting filtrate was designated as the viral lysate-derived dissolved fraction (VDF). The VDF was then stored at −80°C until further use.

### Cell counting of *H. akashiwo* and enumeration of extracellular HaV

To count *H. akashiwo* cells, 1,920 µL of the culture medium was analyzed using RF-500 Flow Cytometer (Sysmex Corporation). Cells were quantified by plotting chlorophyll fluorescence in the red channel (695/50 nm) against forward scatter, and the data were analyzed with FlowJo (Becton, Dickinson and Company) following the manufacturers’ instructions.

For enumerating extracellular HaV particles, 60 mL of the infected *H. akashiwo* culture collected on the first day of the infection experiment (described above) was divided into three equal portions and transferred to three separate flasks. Each culture was then diluted 10-fold with fresh f/2 medium. From each diluted culture, 12 mL was collected daily and centrifuged at 1,500 × *g* for 5 minutes to remove host cells and debris. The supernatant was then centrifuged again under the same conditions. The final supernatant was ultracentrifuged at 104,000 × *g* using an Optima XE-90 Ultracentrifuge (Beckman Coulter Inc.) to pellet the viral particles. Viral DNA was extracted from the pellet using the xanthogenate-SDS method described by Kimura et al. (63). The abundance of HaV103 was assessed by quantifying the gene encoding the major capsid protein (mcp) using quantitative PCR. Primers specific for the mcp gene (HaV53_mcp_290F: 5′-GTCTGAGGACGCACTGTGAA and HaV53_mcp_429R: 5′-GCTGGTACTGCGTACAACGA) were designed from the genome sequence of HaV53 (the GenBank accession number KX008963 (64); . These primers were tested for specific amplification of the target gene using DNA from *H. akashiwo* NIES-293 and HaV103 analysis. PCRs were conducted with TB Green Premix Ex Taq II (TaKaRa Bio) as per the manufacturer’s instruction. The Thermal Cycler Dice Real Time System III (TaKaRa Bio) was used with the following cycling conditions: 1 cycle at 95℃ for 30 s, followed by 40 cycles of 95℃ for 5 s, 60℃ for 30 s, and 72℃ for 15 s. A standard curve for quantifying viral particle abundance was constructed using serial dilutions of known concentrations of HaV103 DNA.

### Measurement of carbon concentration in EDF, IDF, and VDF

To measure the carbon concentration in the VDF, IDF, and EDF, as well as f/2 medium (used as a control), approximately 20 mL of each sample was analyzed using Non-Purgeable Organic Carbon analysis with a Total Organic Carbon Analyzer TOC-L, Shimadzu).

### Setup of microcosm experiments

On 22 June 2022, approximately 10 L of surface seawater (5 m depth) was collected from Osaka Bay, Japan (N 34°19’28”, E 135°7’15”). The seawater was pre-filtered through polycarbonate membrane filters (142 mm diameter, 3.0 µm pore size; Millipore) to remove eukaryotic cells. For the microcosm experiments, 4.8 L of the pre-filtered seawater was again filtered through polycarbonate membrane filters (142 mm diameter, 0.2 µm pore size; Millipore) to isolate coastal prokaryotic cells. The filters capturing the prokaryotic fraction were divided into four equal pieces, and the cells on each piece were resuspended in 300 mL of autoclaved aged seawater in 500 mL flasks. The residual organic matter in the flasks was removed by washing with 6 N HCl followed by Milli-Q water.

VDF was added to three flasks containing the prokaryotic fraction to a final concentration of 80 µmol C/L, which is representative of carbon concentrations observed in natural microalgal blooms (65). The IDF, EDF, and f/2 medium were similarly supplemented to achieve a final concentration of 80 µmol C/L. It should be noted that although the specific chemical compounds were not disclosed by the manufacturer, the artificial seawater we used contained some kind of organic matter. Consequently, the f/2 medium contained 1.19 mmol C/L of organic matter. The flasks were categorized into four treatment groups: control, VDF-treatment, IDF-treatment, and EDF-treatment. Each treatment consisted of three triplicate flasks, designated as replicate flasks I–III. A total of twelve flasks were incubated at 20°C, under the 14.5 hour light/9.5 hour dark cycle at an irradiance of 150 µmol photons m^−2^ s^−1^, for 4 days. These temperature and light conditions represent the typical environmental conditions of bloom-forming seasons at Osaka Bay, Japan.

Control, IDF-treatment, EDF-treatment, and VDF-treatment flasks were homogenized by mixing once daily before subsampling. Each day, 1,920 µL was subsampled from each flask every day and fixed with glutaraldehyde (1% ﬁnal concentration) at 4°C. The fixed prokaryotic cells were stained with SYBR Green I (ﬁnal concentration 1×; Thermo Fisher Science) for 20 minutes at room temperature in the dark. The stained cells were then counted using an S3e Cell Sorter (Bio-Rad) and analyzed using FlowJo (Becton, Dickinson and Company), according to manufacturers’ instructions. Mann–Whitney U tests were performed to compare cell abundances among treatments on the same day.

### DNA extraction and sequencing

For prokaryotic community structure analysis, 1,800 µL of culture medium was subsampled daily from each flask, resulting in a total of 60 samples over 4 days. Prokaryotic cells were collected on polycarbonate membrane filters (25 mm diameter, 0.2 µm pore size; ADVANTEC Toyo Kaisha, Ltd.) and stored at −30°C until DNA extraction. To analyze the prokaryotic community composition in the original seawater, 10 mL of the seawater was filtered on polycarbonate filters (25 mm diameter, 0.2 µm pore size; ADVANTEC Toyo Kaisha, Ltd.), and these filters were stored at −30°C until DNA extraction. DNA was extracted using the method described in our previous study (19). The control flask replicate I sample on day 2 was lost due to a technical error. The 16S rRNA genes were amplified using primers targeting the V3–V4 hypervariable regions, with added overhang adapter sequences at each 5ʹ end, as described by (66) and according to the Illumina 16S sample preparation guide. The amplicons were sequenced with a MiSeq Reagent kit, version 3 (2 × 300  bp read length; Illumina), following the manufacturer’s instruction.

### Sequence processing and ASV generation

From the original seawater sample, 208,405 reads were obtained (Table S1). During the microcosm experiments, the average number of reads per flask was as follows: 1,019,575 for the control, 1,026,863 for the VDF treatment, 1,048,035 for the IDF treatment, and 952,122 for the EDF treatment (Table S1). Quality filtering, denoising, paired-end merging, and construction of the ASV feature table were performed using the DADA2 plugin (67) within QIIME2. Taxonomic assignment of the ASVs was performed using the SILVA (release 138) reference database (68). ASVs assigned to mitochondrial or chloroplast sequences were removed from the feature table and excluded from subsequent analyses. Singleton ASVs were also removed during this process.

### Analysis of community structure and dynamics of prokaryote ASVs

To analyze ASV composition, all sequence data were rarefied to the depth of the lowest sample. The Bray-Curtis dissimilarity index was calculated for pairwise comparisons of the prokaryotic communities using the “vegan” package in R. This index was visualized by principal coordinate analysis (PCoA) with the “stats” package in R. The analysis of similarity (ANOSIM) was performed with the Bray-Curtis dissimilarity scores to assess significance of differences among days or treatments, using the “vegan” package in R. *P*-values for multiple comparisons were corrected using the Bonferroni method.

Relative abundance of each ASV was calculated by dividing the read counts of the ASV by the total read counts of each sample. To estimate the approximate cell density (cells/mL) of each ASV, the relative abundance (ranging from 0 to 1) was multiplied by the total number of prokaryotic cells. ASVs were classified as abundant if they ranked among the top 20 in approximate cell density at least 1 day after day 1 in all the triplicate flasks, and if their maximum approximate cell density was more than twice that at day 0. Differences in the approximate cell density of these ASVs among treatments were tested for statistical significance using LEfSe (69), with treatment as the class and triplicate flasks as the subclass.

### Survey of abundant Osaka Bay ASVs in amplicon data set from Monterey Bay natural bloom samples

To confirm that the ASVs of interest were detected in the natural *H. akashiwo* bloom, we analyzed abundance of these ASVs and *H. akashiwo* during a bloom observed in Monterey Bay, US. The 18S rRNA gene sequence of *H. akashiwo* NIES-293 (DQ470658.1) (70) was obtained from GenBank (71). Raw amplicon reads of 18S rRNA and 16S rRNA gene sequences from natural bloom samples collected daily in Monterey Bay between September 26 and November 16 of 2016 (41) were retrieved from the NCBI Sequence Read Archive (PRJNA533622). These reads were quality-trimmed, merged, and chimeras were removed following the pipeline described in our previous study (19). Samples with fewer than 5,000 quality-controlled reads were excluded from the analysis. The quality-controlled 18S rRNA and 16S rRNA reads were mapped to the NIES-293 sequence and prokaryotic ASVs of interest with 97% and 100% identity, respectively, using VSEARCH (72). Sequences with 97% or higher identity to the 18S rRNA gene of NIES-293 were considered to be *H. akashiwo*. Relative abundance was then calculated using the method described above. Temporal autocorrelation analysis was conducted for both autocorrelation and partial autocorrelation using the *acf* function in R (73). Spearman correlations between *H. akashiwo* and ASVs of interest were calculated based on their relative abundance using the local similarity analysis program (74). A time delay of two sampling intervals (*d* = 2) was permitted. *H. akashiwo*-ASV pairs that met *r* > 0.6 (*P* < 0.01, *q* < 0.05) were regarded as having a significant positive correlation.

### Phylogenetic analysis of abundant ASVs

To determine the phylogenetic positions of the abundant ASVs of interest, nearly complete 16S rRNA gene sequences (> 1,500 bp) of their assigned genera, as identified by Silva (see above), were retrieved from the SILVA NR reference database to construct reference phylogenetic trees. Sequences were aligned using MAFFT (75) with the L-INS-i method, and gap positions were automatically removed using trimAl (76) using the -gappyout option. Phylogenetic tree was then reconstructed using the approximately maximum-likelihood method using FastTree (77). The abundant ASVs of interest were classified onto the reference tree using pplacer (78). Results were visualized with iTOL (79).

### Comparative genomic analysis of abundant ASV relatives

Complete genomes of the closest isolates (100% identity of 16S rRNA sequence) of the ASVs of interest were downloaded from the NCBI RefSeq NR database (80). KEGG modules present in these genomes were identified using METABOLIC (81) with default settings. The presence or absence of each module in each genome was used to analyze the prevalence among ASV-relative lineages.

### Metabolomic analysis using gas chromatography-mass spectrometry (GC-MS)

An aliquot of 100  µL of 2-isopropylmalate (98%; Sigma-Aldrich) was added to the 250 µL each of sodium chloride (26.4  mg mL−1) as the blank, f/2 medium (control), VDF, IDF, and EDF as an internal control. The samples were freeze-dried for over 6  h. To remove residual water, 250 µL of toluene (> 99.7%; FUJIFILM Wako Pure Chemical Corporation) was added to each sample, and the mixture was ultrasonicated for 10  min. The toluene was removed under a gentle flow of N_2_ gas. Metabolite derivatization was performed by adding 80 µL of methoxyamine hydrochloride (98%; Sigma-Aldrich) dissolved in pyridine (20  mg/mL; FUJIFILM Wako Pure Chemical Corporation) to the dried pellet. The mixture was ultrasonicated for 10  min, briefly vortexed to dissolve the pellet, and incubated for 90  min at 30°C with constant rotation at 1,200  rpm in a thermal rotating incubator. Following this, 100 µL of N-methyl-N-trimethylsilyl-trifluoroacetamide (GL Sciences Inc.) was added, and the mixture was ultrasonicated for 10  min, vortexed, and incubated for 30  min at 37°C with constant rotation at 1,2000  rpm. The derivatized mixture was ultrasonicated for an additional 10  min, and residual salts were removed by centrifugation at 16,000  × *g* for 5  min at room temperature. The supernatant was then analyzed by GC-MS.

All derivatized samples were analyzed on GCMS-QP2010Ultra (Shimadzu). Retention times were calibrated with the Qualitative Retention Time Index Standard (AART-STD; Restek Corporation) prior to analysis. The samples were injected onto a DB-5 column (30  cm by 0.25  mm, 1.0 µm film thickness; Agilent). The injector temperature was set at 280°C. Chromatographic separation was achieved with an initial column oven temperature of 100°C (held for 4 minutes), followed by a temperature ramp of 10 °C/min until reaching 320°C, which was then maintained for 11 min. Helium was used as a carrier gas at a constant flow rate of 39  cm/sec. Mass spectra were collected in electron ionization mode at 70 V across the mass range of m/z 45 to 600.

For peak annotation in the GC-MS analysis, 401 compounds from the SHIMADZU Smart Metabolites Database (Shimadzu) were used as references. The relative abundance of each detected compound was calculated by dividing its peak area by that of the internal control. This relative abundance was then normalized to account for the carbon concentration in each sample, allowing for accurate comparison between samples with different carbon concentrations.

## Data Availability

The sample information obtained in this study was deposited in the DNA Data Bank of Japan (DDBJ) under project number PRJDB18787. Raw sequence reads can be found under accession number DRA019308.

## SUPPLEMENTAL MATERIAL

Figure S1 msystems.00816-25-s0001.pdf Shifts in abundance of *H. akashiwo* NIES-293 and *H. akashiwo* virus HaV103 during infection experiments.

Figure S2 msystems.00816-25-s0002.pdf Relative abundance of phylum-level or class-level phylogenetic groups in the microcosm samples.

Figure S3 msystems.00816-25-s0003.pdf UpSet plot indicating the distribution pattern of all the abundant ASVs among the treatments.

Figure S4 msystems.00816-25-s0004.pdf Phylogenetic placement of Vibrionaceae abundant ASVs on reference tree constructed with nearly complete 16S rRNA genes.

Figure S5 msystems.00816-25-s0005.pdf Phylogenetic placement of Pseudoalteromonadaceae abundant ASVs on reference tree constructed with nearly complete 16S rRNA genes.

Figure S6 msystems.00816-25-s0006.pdf Temporal autocorrelation patterns of ASVs that exhibited co-occurring dynamics with *H. akashiwo* in the natural bloom samples.

Supplemental Tables msystems.00816-25-s0007.xlsx Tables S1 to S7.

ASM does not own the copyrights to Supplemental Material that may be linked to, or accessed through, an article. The authors have granted ASM a non-exclusive, world-wide license to publish the Supplemental Material files. Please contact the corresponding author directly for reuse.
