# Supplementary material for: Viral infection to the raphidophycean alga Heterosigma akashiwo affects both intracellular organic matter composition and dynamics of a coastal prokaryotic community
Source: mSystems. 2025 Sep 22;10(10):e00816-25. doi: 10.1128/msystems.00816-25 (PMC12542696; doi:10.1128/msystems.00816-25)
Supplement: Figure S1 — Shifts in abundance of H. akashiwo NIES-293 and H. akashiwo virus HaV103 during infection experiments. [file msystems.00816-25-s0001.pdf]

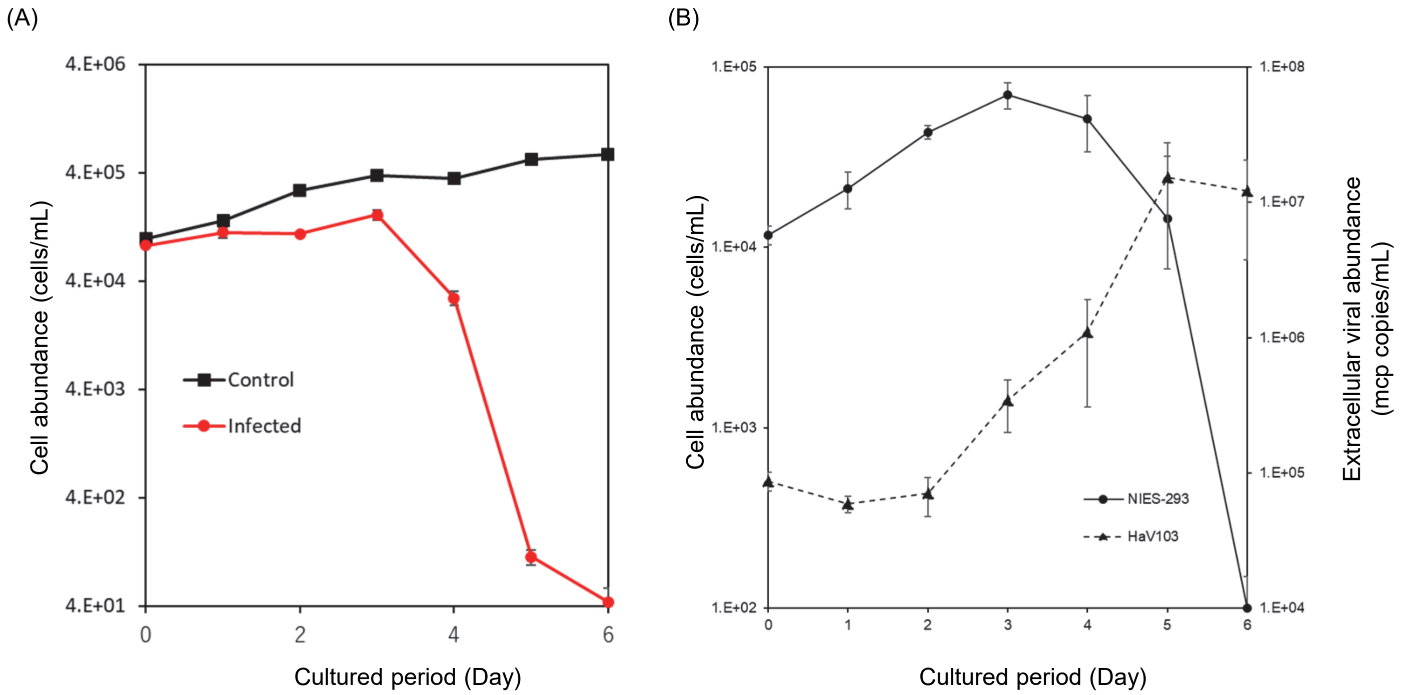

Supplementary Fig. 1. Shifts in abundance of *H. akashiwo* NIES-293 and *H. akashiwo* virus HaV103 during infection experiments. (A) The uninfected control and infected culture. Cell counts were obtained using flow cytometry. The average cell abundance in the triplicate measurements is shown. Error bars indicate standard deviation. (B) 10-times diluted infected culture constructed for monitoring HaV103 abundance. Cell counts were obtained using flow cytometry and HaV103 abundance was analyzed by quantitative PCR targeting major capsid protein gene (*mcp*). The average cell and viral abundance in the triplicate flasks are shown. Error bars indicate standard deviation.
