# Supplementary material for: Viral infection to the raphidophycean alga Heterosigma akashiwo affects both intracellular organic matter composition and dynamics of a coastal prokaryotic community
Source: mSystems. 2025 Sep 22;10(10):e00816-25. doi: 10.1128/msystems.00816-25 (PMC12542696; doi:10.1128/msystems.00816-25)
Supplement: Figure S2 — Relative abundance of phylum-level or class-level phylogenetic groups in the microcosm samples. [file msystems.00816-25-s0002.pdf]

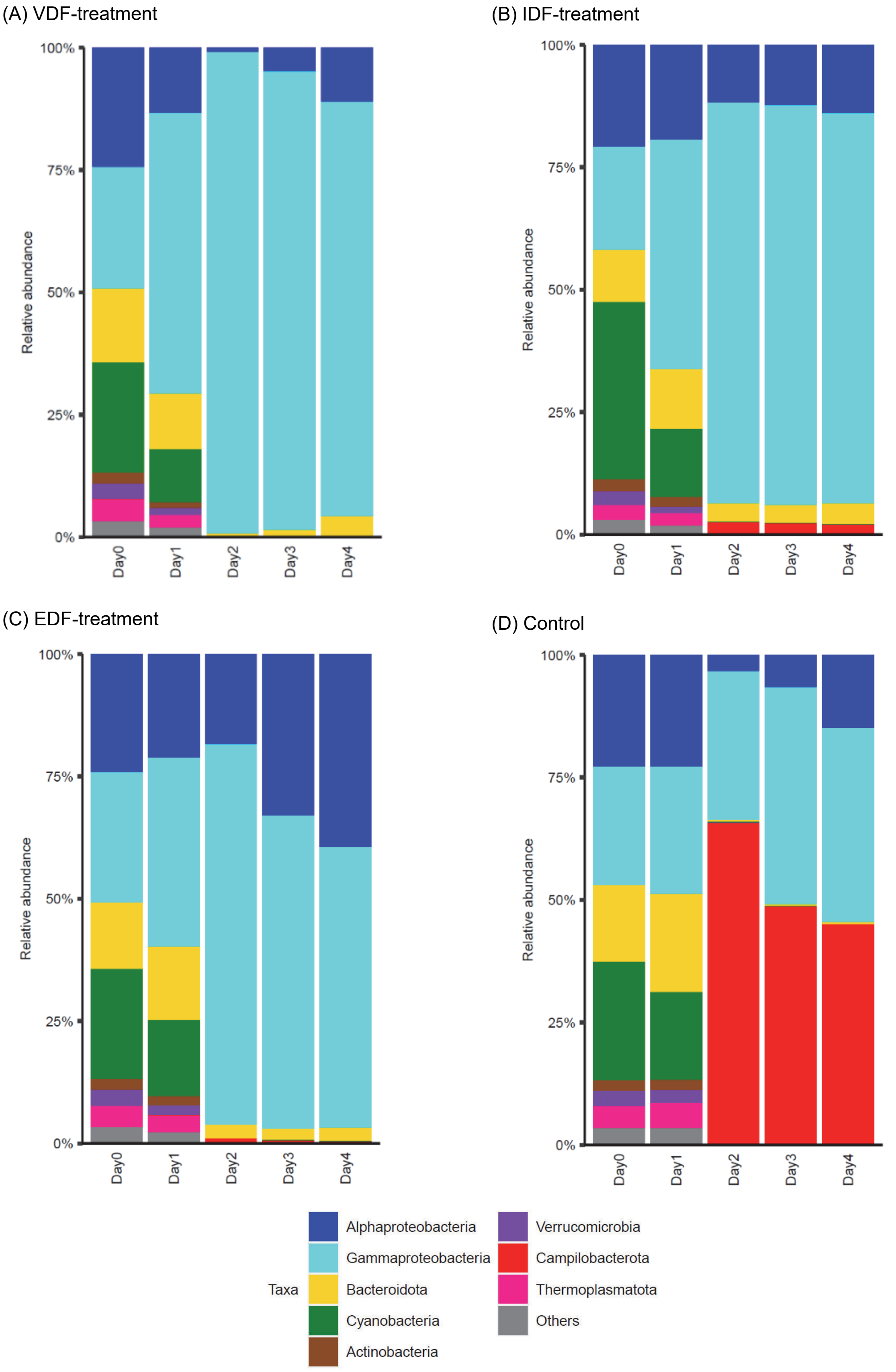

Supplementary Fig. 2. Relative abundance of phylum-level (class-level for proteobacteria) phylogenetic groups in the microcosm samples. For each treatment, averaged relative abundance in the triplicate flasks is shown.
