# Supplementary material for: Viral infection to the raphidophycean alga Heterosigma akashiwo affects both intracellular organic matter composition and dynamics of a coastal prokaryotic community
Source: mSystems. 2025 Sep 22;10(10):e00816-25. doi: 10.1128/msystems.00816-25 (PMC12542696; doi:10.1128/msystems.00816-25)
Supplement: Figure S3 — UpSet plot indicating the distribution pattern of all the abundant ASVs among the treatments. [file msystems.00816-25-s0003.pdf]

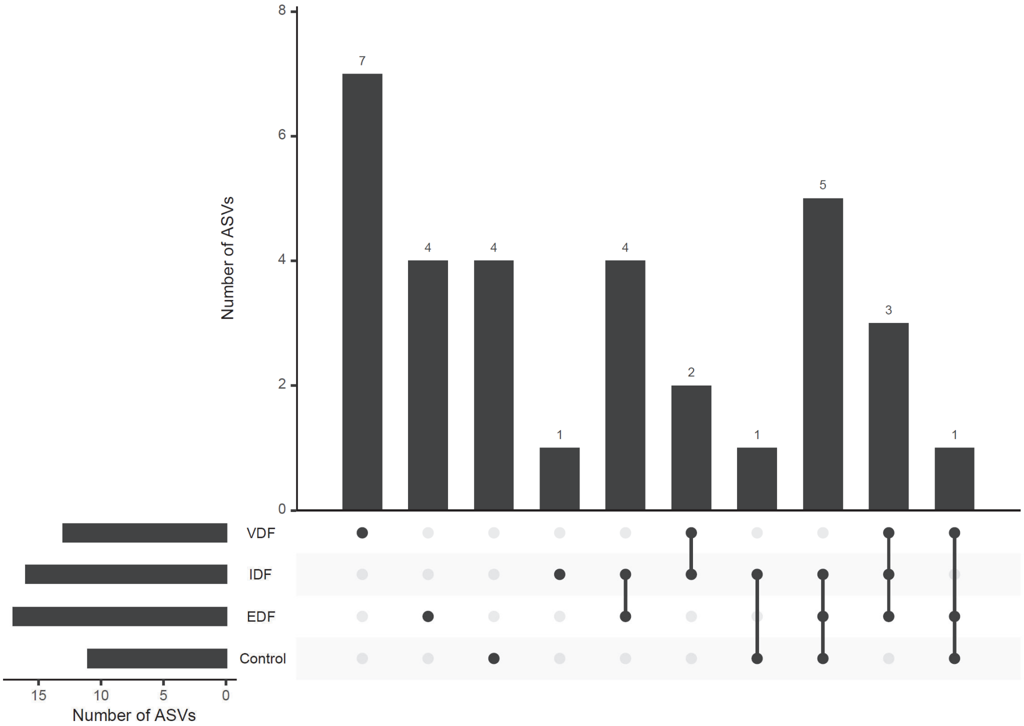

Supplementary Fig. 3. UpSet plot indicating the distribution pattern of all the abundant ASVs among the treatments. The number shown above bar graph indicates those of abundant ASVs detected in each treatment.
