# Supplementary material for: Viral infection to the raphidophycean alga Heterosigma akashiwo affects both intracellular organic matter composition and dynamics of a coastal prokaryotic community
Source: mSystems. 2025 Sep 22;10(10):e00816-25. doi: 10.1128/msystems.00816-25 (PMC12542696; doi:10.1128/msystems.00816-25)
Supplement: Figure S4 — Phylogenetic placement of Vibrionaceae abundant ASVs on reference tree constructed with nearly complete 16S rRNA genes. [file msystems.00816-25-s0004.pdf]

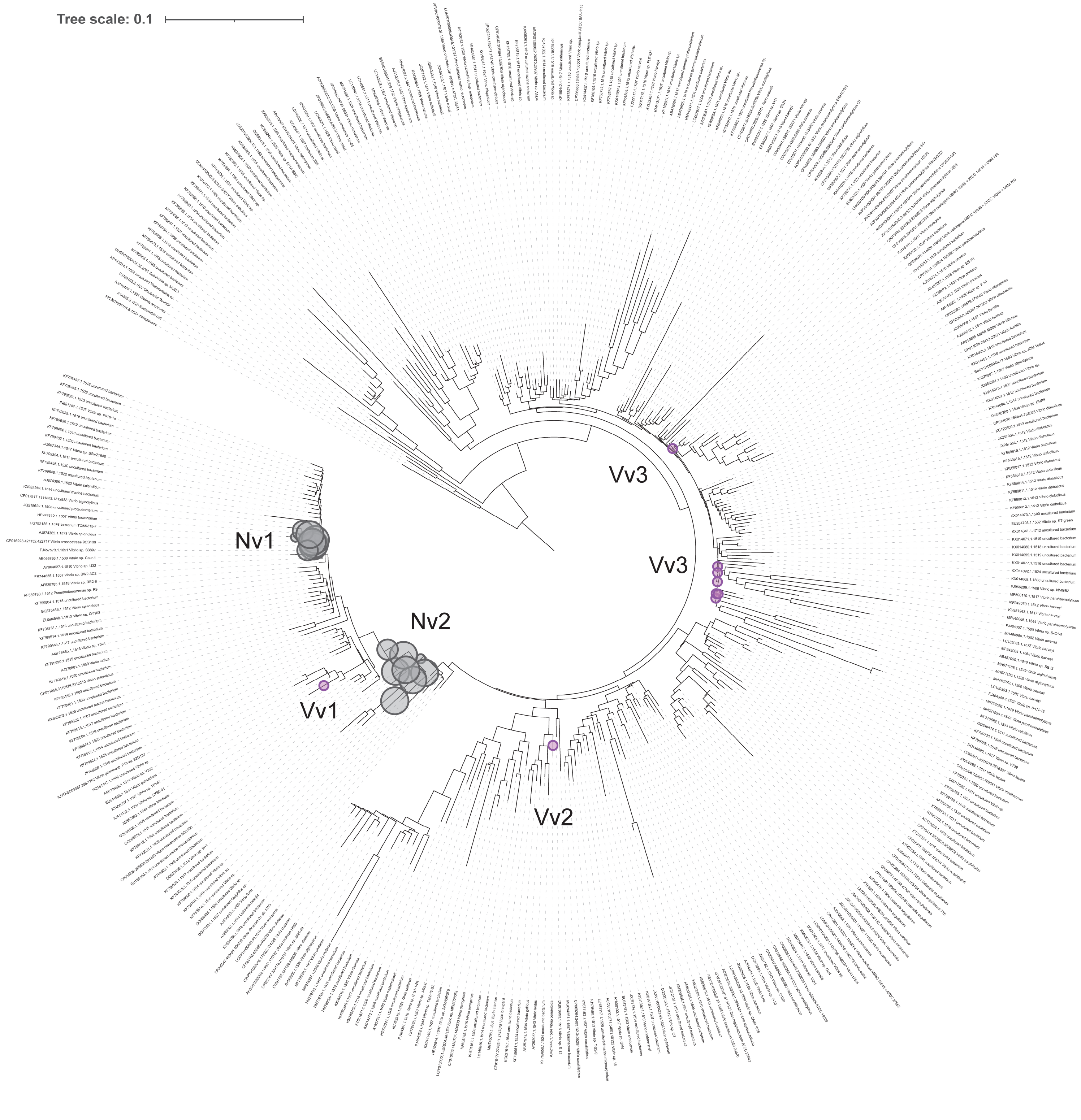

Supplementary Fig. 4. Phylogenetic placement of Vibrionaceae abundant ASVs on reference tree constructed with nearly complete 16S rRNA genes. The reference phylogenetic trees were constructed using the approximately-maximum likelihood method. The purple circles indicate the nodes where VDF-specific ASVs were mapped and the gray circles did the nodes where VDF-nonspecific ASVs were mapped. The size of each circle represents the confidence of the placement for each ASV sequence; larger circles indicate higher likelihoods. Vv1; ASV_3626. Vv2; ASV_3968. Vv3; ASV_4145. Nv1; ASV_516, ASV_694, and ASV_1745. Nv2; ASV_208, ASV_3278, and ASV_1745.
