# Supplementary material for: Viral infection to the raphidophycean alga Heterosigma akashiwo affects both intracellular organic matter composition and dynamics of a coastal prokaryotic community
Source: mSystems. 2025 Sep 22;10(10):e00816-25. doi: 10.1128/msystems.00816-25 (PMC12542696; doi:10.1128/msystems.00816-25)
Supplement: Figure S5 — Phylogenetic placement of Pseudoalteromonadaceae abundant ASVs on reference tree constructed with nearly complete 16S rRNA genes. [file msystems.00816-25-s0005.pdf]

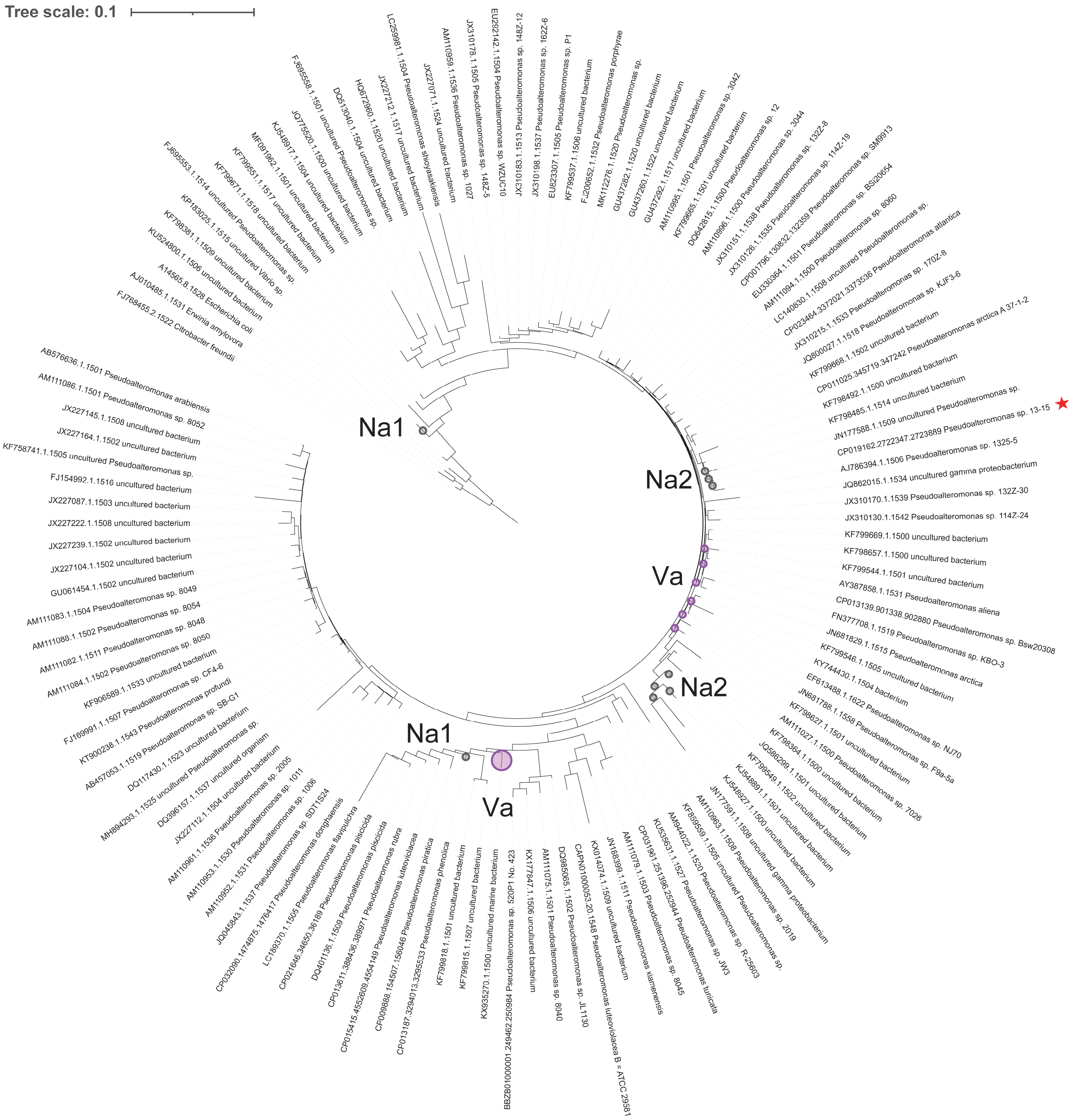

Supplementary Fig. 5. Phylogenetic placement of Pseudoalteromonadaceae abundant ASVs on reference tree constructed with nearly complete 16S rRNA genes. The reference phylogenetic trees were constructed using the approximately-maximum likelihood method. The purple circles indicate the nodes where VDF-specific ASVs were mapped and the gray circles did the nodes where VDF-nonspecific ASVs were mapped. The size of each circle represents the confidence of the placement for each ASV sequence; larger circles indicate higher likelihoods. Va; ASV_1731 and ASV_4400. Na1; ASV_927. Na2; ASV_335. The sequence CP019162.2722347.2723889_*Pseudoalteromonas*_sp._13-15, indicated by the red star, clustered with the 16S rRNA gene sequence of *P. marina* (CP023558) with >99.7% sequence identity.
