# Supplementary material for: Viral infection to the raphidophycean alga Heterosigma akashiwo affects both intracellular organic matter composition and dynamics of a coastal prokaryotic community
Source: mSystems. 2025 Sep 22;10(10):e00816-25. doi: 10.1128/msystems.00816-25 (PMC12542696; doi:10.1128/msystems.00816-25)
Supplement: Figure S6 — Temporal autocorrelation patterns of ASVs that exhibited co-occurring dynamics with H. akashiwo in the natural bloom samples. [file msystems.00816-25-s0006.pdf]

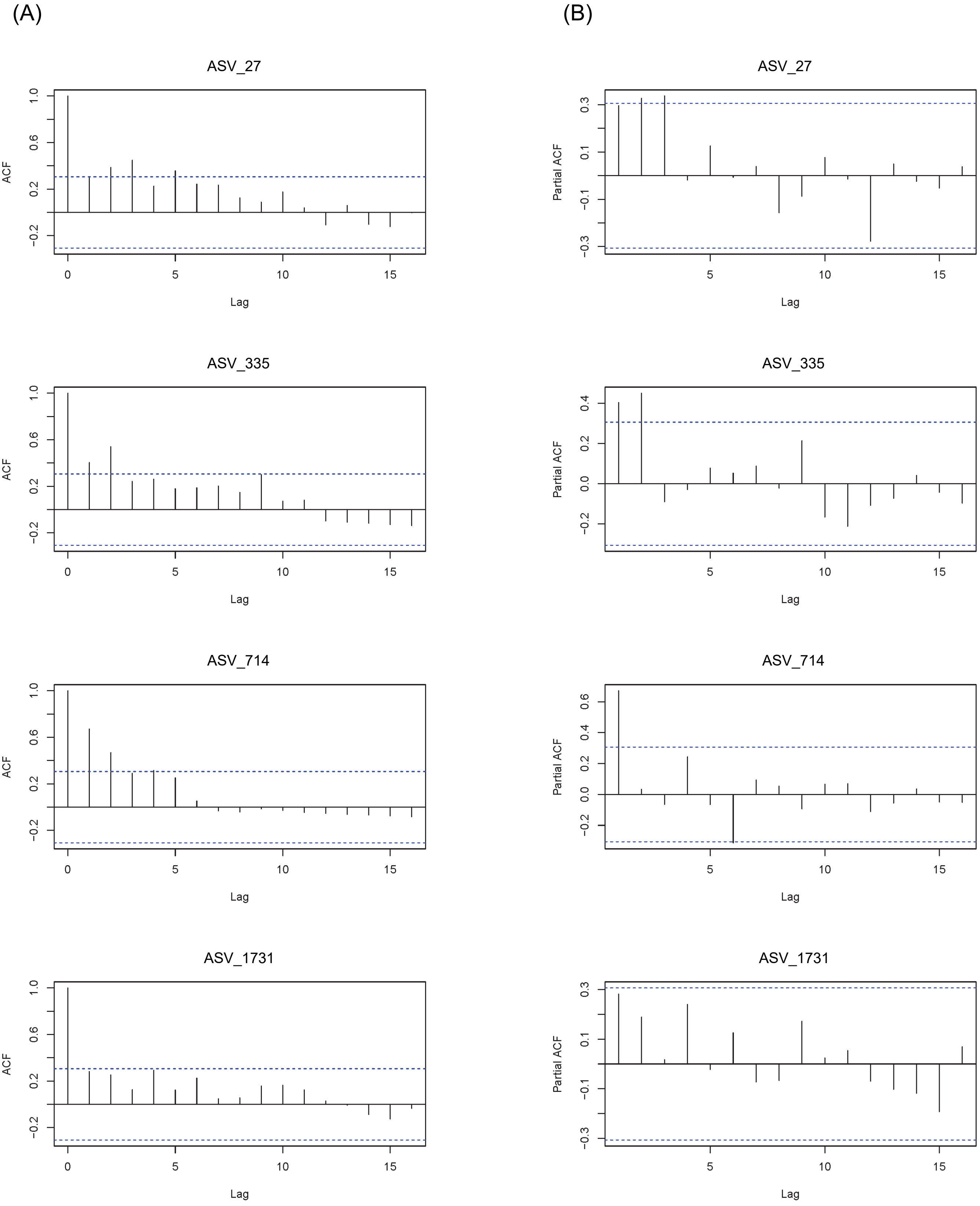


**Supplementary Fig. 6.** Temporal autocorrelation patterns of ASVs that exhibited co-occurring dynamics with *H. akashiwo* in the natural bloom samples. (A) autocorrelation coefficients. (B) partial autocorrelation coefficients. The microbiome sequence datasets almost daily collected between September 26–November 16, 2016 (Nowinski *et al.,* 2019) were used. Relative abundance of *H. akashiwo* NIES-293 close relatives was calculated by mapping quality-controlled reads of 18S rRNA genes to the NIES-293 sequence with 97% identity using VSEARCH. Relative abundance of each ASV was calculated by mapping quality-controlled reads of 16S rRNA genes to the ASV sequence with 100% identity using VSEARCH. The autocorrelation and partial autocorrelation coefficients were analyzed based on the relative abundance of ASV of interest using the *acf* function in R. ASVs that showed positive correlation with the dynamics of *H. akashiwo* NIES-293 close relatives are shown (Spearman correlation; $r > 0.6$, $p < 0.01$ and $Q < 0.05$).
